# Discovery of newborn Wilson disease biomarkers via integrated next-generation sequencing and untargeted metabolomics

**DOI:** 10.1186/s13023-025-04173-6

**Published:** 2025-12-26

**Authors:** Xianwei Guan, Yun Sun, Yanyun Wang, Yahong Li, Zhilei Zhang, Dongyang Hong, Peiying Yang, Xiaowei Liang, Xin Wang, Bin Yu

**Affiliations:** 1 Genetic Medicine Center, Women’s Hospital of Nanjing Medical University, Nanjing Women and Children’s Healthcare Hospital, 123 Tianfei St. Qinhuai District, Nanjing, 210004 China; 2 Department of Medical Genetics, Changzhou Maternal and Child Health Care Hospital, Changzhou Medical Center of Nanjing Medical University, 16 Dingxiang St. Zhonglou District, Changzhou, 213000 China

**Keywords:** Wilson’s disease, Differential metabolites, Liver damage, Neurological symptoms, Tyrosine metabolism

## Abstract

**Background:**

Wilson disease (WD) is an autosomal recessive disorder caused by variants in the ATP7B gene, leading to copper metabolism dysfunction and multi-organ damage. Early diagnosis is critical for improving clinical outcomes, but current screening methods have limitations. Metabolomics can reveal early metabolic disturbances in disease; however, the metabolic profile of newborns with WD remains unexplored. This study aimed to identify potential metabolic biomarkers for early WD detection through untargeted metabolomic analysis.

**Methods:**

Dried blood spot (DBS) samples from six genetically confirmed WD positive newborns and 84 healthy controls were analyzed using liquid chromatography-mass spectrometry (LC-MS). Multivariate statistical analysis was employed to identify differentially abundant metabolites. Pathway enrichment analysis and receiver operating characteristic (ROC) curve evaluation were performed to assess diagnostic performance.

**Results:**

A total of 29 significantly altered metabolites (21 upregulated, 8 downregulated) were identified in WD positive newborns, primarily associated with tyrosine metabolism. ROC analysis revealed 11 metabolites with an area under the curve (AUC) > 90%. Additionally, two pairs of isomers also demonstrated exhibited high diagnostic sensitivity and specificity and were closely linked to WD pathogenesis. In positive group, tyrosine metabolism pathway was most significantly affected, as evidenced by increased levels of 3,4-Dihydroxyphenylacetic acid and Homogentisic acid, alongside a decreased level of Gentisaldehyde.

**Conclusion:**

WD positive newborns exhibit distinct metabolic reprogramming prior to copper accumulation, with tyrosine metabolism dysregulation as a potential early feature. The identified differential metabolites may serve as promising biomarkers for newborn WD screening, providing a foundation for metabolomics-based early diagnostic strategies.

**Supplementary Information:**

The online version contains supplementary material available at 10.1186/s13023-025-04173-6.

## Introduction

Wilson disease (WD), or hepatolenticular degeneration, is an autosomal recessive disorder of copper metabolism caused by variants in the ATP7B gene. First described by British neurologist Samuel Alexander Kinnier Wilson in 1912 [1], WD results from impaired function of the copper-transporting P-type ATPase encoded by ATP7B. This defect prevents copper from being properly incorporated into ceruloplasmin (Cp) in the Golgi apparatus of hepatocytes, leading to systemic copper accumulation. As copper deposits in extrahepatic tissues such as the brain, cornea, kidneys, bones, and joints, patients may develop neurological symptoms, Kayser-Fleischer (K-F) rings, proteinuria, anemia, renal dysfunction, and osteoarticular disease, all of which significantly affect quality of life [2]. The clinical manifestations of WD are heterogeneous and are commonly classified into hepatic, neurologic, mixed, and other forms. In infants and children, the hepatic type predominates due to early hepatic copper deposition. Some pediatric patients may experience acute liver failure accompanied by hemolytic anemia, which if left untreated, can be fatal in up to 90% of cases [3]. Neurological symptoms typically occur after hepatic involvement and may include dystonia, tremor, bradykinesia, and behavioral disturbances; in severe cases, psychosis or suicidal tendencies may arise [4, 5]. Historically, the prevalence of WD has been estimated at 1 in 30,000, with a carrier frequency of approximately 1 in 90. However, these figures have been increasingly questioned [6, 7]. Recent meta-analyses and genomic data from newborn screening programs in China suggest a much higher genetic prevalence, estimated at 1 in 7,194 [8, 9]. This implies that WD may be underdiagnosed or misdiagnosed in clinical practice. As one of the few treatable inherited metabolic disorders, early, individualized, and lifelong therapy is the mainstay of WD management [10]. Without timely intervention, patients may progress to severe disability or death, with a reported mortality rate of 5–6.1% [11], most often due to delayed diagnosis [12]. WD typically manifests before the age of 40 [13], with the youngest reported case diagnosed at just 8 months of age [14]. The neurologic form is often associated with rapid disease progression and irreversible damage at diagnosis [15]. In contrast, early detection and treatment can significantly improve prognosis [14, 16], underscoring the importance of early identification.

Some experts recommend Cp testing at 3 years old as the optimal time for WD screening [17]. However, collecting dried blood spot (DBS) or urine samples from children at this age poses logistical challenges, making large-scale screening difficult and increasing the likelihood of missed diagnoses. Integrating WD into newborn screening programs may offer a more feasible solution. Nevertheless, studies have shown that newborn Cp testing may subject to various confounding factors—including ATP7B carrier status, hepatic or renal impairment, estrogen level fluctuations, and incomplete sample elution—leading to considerable false-positive and false-negative rates that compromise its reliability in ruling out Wilson disease [17, 18]. Moreover, immunoturbidimetric detection of Cp in DBS requires at least three 3.2 mm discs to achieve sufficient sensitivity [19], imposing significant sample volume requirements. Although copper metabolism dysregulation is a hallmark of WD, serum copper levels are inconsistent—often decreased due to deficiency in holo-ceruloplasmin, yet sometimes falling within the normal range [20]. Furthermore, no commercially available kits currently support copper quantification in DBS. Other copper metabolism indicators—such as 24-hour urinary copper, non-ceruloplasmin-bound copper, exchangeable copper, and hepatic copper content—only become abnormal once copper overload is advanced, rendering them unsuitable for early screening in newborns. In recent years, next-generation sequencing (NGS)-based expanded genomic newborn screening programs, including the ATP7B gene, have shown promising results both in China and abroad [9, 21, 22], highlighting NGS as a potentially powerful tool for early WD detection. However, limitations remain, including missed pathogenic variants [23], unclear genotype-phenotype correlations [15, 24], high cost, technical complexity, and challenges in variant interpretation. Therefore, it is essential to develop reliable, efficient, and clinically scalable screening strategies and novel biomarkers for newborn WD detection.

Metabolomics, particularly when based on liquid chromatography platforms, enables high-throughput detection of thousands of metabolites in biological samples such as serum or DBS. It reflects the true metabolic state of the body and can sensitively detect even subtle metabolic abnormalities [25]. After birth, the metabolic patterns of newborn undergo significant changes. Characterizing newborn’s metabolic profile and understanding the metabolic alterations caused by various factors hold great importance for nutritional support, growth and development, disease treatment, and prognosis during the neonatal period [26]. In recent years, numerous researchers have utilized metabolomic techniques to study diseases such as pediatric acute myeloid leukemia [27], infantile hypertrophic pyloric stenosis [28], Autism [29], Maple Syrup Urine Disease [30] through DBS from newborns, yielding promising findings. By constructing disease-specific metabolic profiles, metabolomics allows the identification of differential metabolites associated with pathogenesis, offering a promising approach to discover novel biochemical biomarkers for disease screening.

Therefore, this study aims to integrate untargeted metabolomic analysis with genomic newborn screening to investigate dried blood spot samples from both WD-positive and healthy newborns. Our goal is to identify WD-related metabolic alterations and explore potential biochemical biomarkers for use in newborn WD screening.

## Materials and methods

### Participants and sample collection

Between March 18, 2022, and December 31, 2023, a total of 26,155 newborns delivered at Nanjing Maternity and Child Health Care Hospital were enrolled. Heel blood was collected from each newborn within 48 h after birth to prepare dried blood spot (DBS) filter papers (S&S 903, manufactured by Guizhou Leading Co., Ltd.), which complies with the standards of the Clinical and Laboratory Standards Institute. The DBS samples were stored at -20 °C and < 30% humidity until the assay was performed. Newborn genomic screening, including analysis of the ATP7B gene, was performed using next-generation sequencing (NGS). For newborns in whom two or more variants were detected in the ATP7B gene, parental segregation analysis was conducted. If the variants were confirmed to be located on different parental chromosomes (i.e., in trans), the newborn was classified as genetically confirmed WD positive (hereafter referred to as “WD positive newborns”).

Ethical approval for this study was obtained from the Ethics Committee of the Women’s Hospital of Nanjing Medical University (approval number: 2021KY-071). Written informed consent was obtained from the parents or legal guardians of all participating newborns.

### Metabolite extraction from DBS samples for untargeted metabolomics

From each fully saturated dried blood spot [31], a 3.2 mm disc was punched utilizing a Panthera-Puncher™ 9 system (PerkinElmer). A single puncher head was employed for all samples to maintain processing uniformity. The discs were subsequently transferred into individual 1.5 mL microcentrifuge tubes. After the addition of 100 µL pure water and 30-minute sonication in an ice-water bath, 400µL extraction solution (without internal standards) was added, and the mixture was vortex-mixed for 30 s, sonicated in a 4 °C water bath for 10 min, and incubated at -40 °C for 1 h to precipitate proteins. Samples were then centrifuged at 12,000 rpm (RCF = 13,800 × g, rotor radius = 8.6 cm) for 15 min at 4 °C. After drying a 400 µL supernatant aliquot, 100 µL of extraction solution (methanol: acetonitrile: water = 2:2:1, v/v/v) with isotope-labeled internal standards was added for reconstitution. The sample was then sonicated (10 min) and centrifuged (12,000 rpm, 15 min). The resulting supernatant was transferred to a clean glass vial for metabolomic analysis. A pooled quality control (QC) sample was prepared by mixing equal aliquots of the supernatants from all samples, QC samples and study samples received identical treatment.

### LC-MS/MS analysis

For polar metabolites, LC-MS/MS analyses were performed using an UHPLC system (Vanquish, Thermo Fisher Scientific) with a Waters ACQUITY UPLC BEH Amide (2.1 mm × 50 mm, 1.7 μm) coupled to Orbitrap Exploris 120 mass spectrometer (Orbitrap MS, Thermo). The mobile phase consisted of 25 mmol/L ammonium acetate and 25 mmol/L ammonia hydroxide in water(pH = 9.75)(A) and acetonitrile (B). The auto-sampler temperature was 4 ℃, and the injection volume was 2 µL. The Orbitrap Exploris 120 mass spectrometer was used for its ability to acquire MS/MS spectra on information-dependent acquisition (IDA) mode in the control of the acquisition software (Xcalibur, Thermo). In this mode, the acquisition software continuously evaluates the full scan MS spectrum. The ESI source conditions were set as following: sheath gas flow rate as 50 Arb, Aux gas flow rate as 15 Arb, capillary temperature 320 ℃, full MS resolution as 60,000, MS/MS resolution as 15,000, collision energy: SNCE 20/30/40, spray voltage as 3.8 kV (positive) or -3.4 kV (negative), respectively.

The system equilibration sequence consisted of 5 blank injections followed by 3 balance quality control (BQC) injections. To minimize technical artifacts and batch effects, a randomized injection sequence was implemented for all study samples, QC samples and blank samples were incorporated at intervals throughout the process.

### Data management procedures and metabolite identification

To mitigate technical variability and enhance the biological interpretability of the results, a comprehensive data preprocessing workflow was applied to the raw metabolomic data. This included outlier removal, filtering of missing values, data imputation, and normalization.

Metabolite identification was performed by matching experimental data against reference libraries using key parameters including retention time (RT), precursor mass-to-charge ratio (MS1), and MS/MS fragmentation patterns (MS2) [32]. Identification confidence was classified into four levels according to established metabolomics reporting standards:

Level 1: Metabolites in the sample show matched MS1, MS2 and RT with authentic standards.

Level 2: Metabolites in the sample show matched MS1 and MS2 spectra with public databases.

Level 3: Metabolites in the sample show matched MS1, MS2 and RT with putative compounds.

Level 4: Unknown compounds.

### Data preprocessing and annotation

The raw data were converted to the mzXML format using ProteoWizard and processed with an in-house program. which was developed using R and based on XCMS, for peak detection, extraction, alignment, and integration. The R package and the BiotreeDB(V3.0) were applied in metabolite identification [33]. The software used for metabolite identification included: XCMS (v3.12.0) and MetDNA2 (v1.4.5.92). Commonly used parameters were a signal-to-noise ratio (snthresh) of 3, a minimum peak width (mzdiff) of 0.01, and an absolute noise level (noise) of 0. Orthogonal Projections to Latent Structures- Discriminant Analysis (OPLS-DA) was used for assessment of outliers and supervised clustering. Data were normalized by the sum of theintensity of metabolites, auto-scaled, and then log2-transformed for analysis. Missing values were detected and replaced by the half of the minimum value. The Benjamin-Hochberg false discovery rate (FDR) method was used to address multiple comparisons issue, and the significance threshold of all statistical analyses in our study is corrected Q value less than 0.05. Metabolites with a Q-value < 0.05 and (Variable important projection, VIP) > 1 were considered to be differentially abundant between the groups. Commercial databases including KEGG (http://www.genome.jp/kegg/) and MetaboAnalyst (http://www.metaboanalyst.ca/) were used for pathway enrichment analysis, these two databases were accessed on June 16, 2025. Statistical analyses were performed using R software version 4.1 and SPSS software version 17.0.

## Results

### Baseline characteristics of WD positive and healthy newborns

A total of six WD positive newborns were included in this study. In addition, 84 healthy newborns were selected as the control group, consisting of 54 carriers with only one identified ATP7B variant or two variants from the same chromosomes (i.e., in cis) (Table S1) and 30 negative newborns with no pathogenic variants detected in ATP7B. These 30 negative newborns consisted of 10 subjects each from the 2022, 2023, and 2024 birth cohorts. Genotypes and follow-up results of the WD positive group are summarized in Table 1. Basic demographic and clinical characteristics of the two groups are shown in Table S2. There were no significant differences in sex, mode of delivery, birth weight, or gestational age between the two groups (*P* > 0.05).

**Table 1:** WD positive newborns characteristics

No.	Birth date	Gender	Paternal variant	Maternal variant	Follow-up
1	2022.4.20	male	c.3316G > A, c.588 C > A	c.2333G > T	1y: Normal LFTs/Cp, asymptomatic
2	2022.7.2	female	c.3188 C > T	c.3741 C > G	1y: 1.5y, 2y: Normal LFTs (Cp NA), asymptomatic
3	2022.7.27	male	c.2297 C > T	c.2804 C > T	2y: Normal LFTs/US, low Cp, asymptomatic
4	2023.3.1	male	c.2333G > T	c.2975 C > T	8mo: Normal LFTs, low Cp, asymptomatic
5	2023.6.21	male	c.2333G > T	c.2463_2464insC	1.5y: Elevated ALT (normal MRI), high 24 h-urine Cu, low Cp → started Zn/UDCA; 2y: Persistent abnormal LFTs
6	2023.11.21	male	c.2333G > T	c.3700-1G > A	1.5y: Normal LFTs/Cp, asymptomatic

y = year, mo = month, LFTs = liver function tests, ALT = alanine aminotransferase, US = ultrasound, MRI = magnetic resonance imaging, Cp = ceruloplasmin, Cu = copper, Zn = zinc, UDCA = ursodeoxycholic acid, NA = not assessed

### General metabolic characteristics of the two groups

**Fig. 1:**
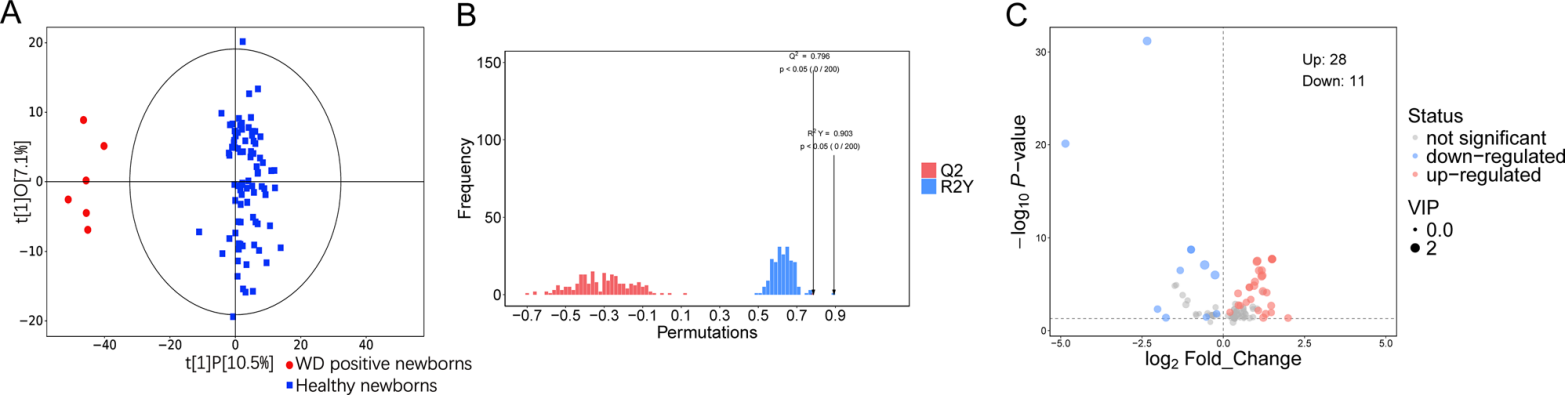
General metabolic characteristics of two groups. (**A**) OPLS-DA score scatter plots of metabolomics data in two groups. (**B**) Permutation histogram of the original OPLS-DA with a positive model, strongly indicating that the original model is valid and shows signs of no overfitting. The permutation test was repeated 200 times in the cross-validation plot. (**C**) Volcano plot representation indicating the -log10 (P-value) and log2 (fold-change) of individual blood metabolic ion features of WD positive newborns compared to healthy newborns group (Q-value < 0.05, VIP > 1). OPLS-DA, Orthogonal Projections to Latent Structures- Discriminant Analysis; VIP, Variable Importance in the Projection

All samples were subjected to metabolomic analysis in a single batch after the complete sample collection. Throughout the experimental process, the interspersed QC samples and blank samples both demonstrated satisfactory stability and all QC results were within the controlled range (Fig. S1, Table S3). Orthogonal partial least squares discriminant analysis (OPLS-DA) was performed to assess the overall metabolic profiles of WD positive and healthy newborns. No outliers were observed based on the sample distribution. The OPLS-DA score plot revealed partial but evident separation between the two groups, indicating distinguishable metabolic profiles (Fig. 1A). A permutation test (*n* = 200) confirmed the robustness and predictive ability of the original model, as the Q² and R²Y values of the original model (Q² = 0.796, *P* < 0.05; R²Y = 0.903, *P* < 0.05) were significantly higher than those of the permuted models (Fig. 1B), and no overfitting was detected. After false discovery rate (FDR) correction, 450 significantly different metabolites (Q-value < 0.05, VIP > 1) were identified between the two groups, of which 39 were successfully annotated (Fig. 1C, Table S4).

### Differential metabolites between WD and NC groups

**Fig. 2:**
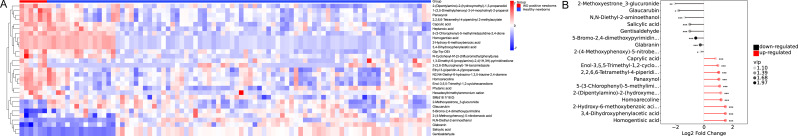
Differences of metabolomic profiles between WD positive newborns and healthy newborns. (**A**) Heat map of differential metabolites between Positive newborns and healthy newborns. Rows, metabolites; columns, newborn samples. Red and blue color intensity indicates increased and reduced abundance. (**B**) Matchstick of downregulated metabolites and top 10 upregulated metabolites between WD positive newborns and healthy newborns. Calculate the inter group ratio by quantifying the differential metabolites and perform a logarithmic transformation with a base of 2. The horizontal axis displays the multiple of logarithmic transformation, and the color depth of the dots represents the size of the VIP value. *, 0.01 < *P* < 0.05; **, 0.001 < *P* < 0.01; ***, *P* < 0.001

After excluding metabolites found to be unstable over storage time (Fig. S2) and exogenous metabolites, 29 metabolites still exhibited significant between two groups (Table 2). Among them, 21 were upregulated and 8 were downregulated in the WD group (Fig. 2A). These metabolites mainly included organic acids and derivatives, aromatic compounds, lipids and lipid-like molecules, and oxygen-containing organic compounds. Notably upregulated metabolites (fold change > 2.5) in the WD group included 2-Hydroxy-6-methoxybenzoic acid, 3,4-Dihydroxyphenylacetic acid, Homogentisic acid, Hexadecyltrimethylammonium cation, 2-(2,6-Difluorophenyl)-1 H-benzimidazole, and N-Cyclohexyl-N’-[3-(trifluoromethyl)phenyl]urea. Metabolites significantly downregulated (fold change < 0.5) included Salicylic acid, Gentisaldehyde, N, N-Diethyl-2-aminoethanol, 2-Methoxyestrone-3-glucuronide, and Glaucarubin (Fig. 2B; Table 2). Among them, pairs such as 2-Hydroxy-6-methoxybenzoic acid, 3,4-Dihydroxyphenylacetic acid, and Homogentisic acid (No. 1–3); Salicylic acid and Gentisaldehyde (No. 22–23) were identified as isomers.

**Table 2:** Differentially abundant metabolites in WD positive newborns compared to healthy newborns

No.	Metabolite	VIP	FC	log2.FC	*p*-value	FDR
**Increased abundance**						
1	2-Hydroxy-6-methoxybenzoic acid	1.45	2.83	1.50	< 0.001	< 0.001
2	3,4-Dihydroxyphenylacetic acid	1.45	2.83	1.50	< 0.001	< 0.001
3	Homogentisic acid	1.45	2.83	1.50	< 0.001	< 0.001
4	Panaxynol	1.49	2.07	1.05	< 0.001	< 0.001
5	2,2,6,6-Tetramethyl-4-piperidinyl 2-methylacrylate	1.54	2.04	1.03	< 0.001	< 0.001
6	2-(Dipentylamino)-2-(hydroxymethyl)-1,3-propanediol	1.32	2.27	1.18	< 0.001	< 0.001
7	5-(3-Chlorophenyl)-5-methylimidazolidine-2,4-dione	1.44	2.15	1.10	< 0.001	< 0.001
8	Homoarecoline	1.51	2.29	1.20	< 0.001	< 0.001
9	Enol-3,5,5-Trimethyl-1,2-cyclohexanedione	1.33	1.96	0.97	< 0.001	< 0.001
10	Caprylic acid	1.17	1.74	0.80	< 0.001	< 0.001
11	Phytanic acid	1.24	1.37	0.45	< 0.001	< 0.001
12	N2,N4-Diethyl-6-hydrazino-1,3,5-triazine-2,4-diamine	1.07	1.80	0.85	< 0.001	< 0.01
13	Heptanoic_acid	1.16	1.38	0.46	< 0.01	< 0.01
14	Ethyl 3-(piperidin-4-yl)propanoate	1.09	1.43	0.52	< 0.01	< 0.01
15	Hexadecyltrimethylammonium cation	1.24	2.78	1.47	< 0.01	< 0.01
16	1,3-Dimethyl-6-(propylamino)-2,4(1 H,3 H)-pyrimidinedione	1.11	2.11	1.08	< 0.01	< 0.05
17	SM(d18:1/18:0)	1.02	1.15	0.20	< 0.05	< 0.05
18	2-(2,6-Difluorophenyl)-1 H-benzimidazole	1.05	2.78	1.47	< 0.05	< 0.05
19	1-(2,5-Dimethylphenoxy)-3-(4-morpholinyl)-2-propanol	1.30	2.47	1.31	< 0.05	< 0.05
20	Glp-Trp-OEt	1.38	2.35	1.23	< 0.05	< 0.05
21	N-Cyclohexyl-N’-[3-(trifluoromethyl)phenyl]urea	1.15	3.98	1.99	< 0.05	< 0.05
**Decreased abundance**						
22	Salicylic acid	1.32	0.50	-0.99	< 0.001	< 0.001
23	Gentisaldehyde	1.32	0.50	-0.99	< 0.001	< 0.001
24	5-Bromo-2,4-dimethoxypyrimidine	1.97	0.67	-0.57	< 0.001	< 0.001
25	N, N-Diethyl-2-aminoethanol	1.10	0.40	-1.33	< 0.001	< 0.001
26	Glabranin	1.70	0.84	-0.25	< 0.001	< 0.001
27	2-Methoxyestrone_3-glucuronide	1.11	0.25	-2.02	< 0.01	< 0.05
28	2-(4-Methoxyphenoxy)-5-nitrobenzoic acid	1.10	0.87	-0.21	< 0.05	< 0.05
29	Glaucarubin	1.25	0.29	-1.77	< 0.05	< 0.05

Metabolites are ranked by statistical significance (smallest P-value first). VIP, Variable important projection; FC, Fold change; FDR, False discovery rate

### Pathway enrichment analysis of differential metabolites

**Fig. 3:**
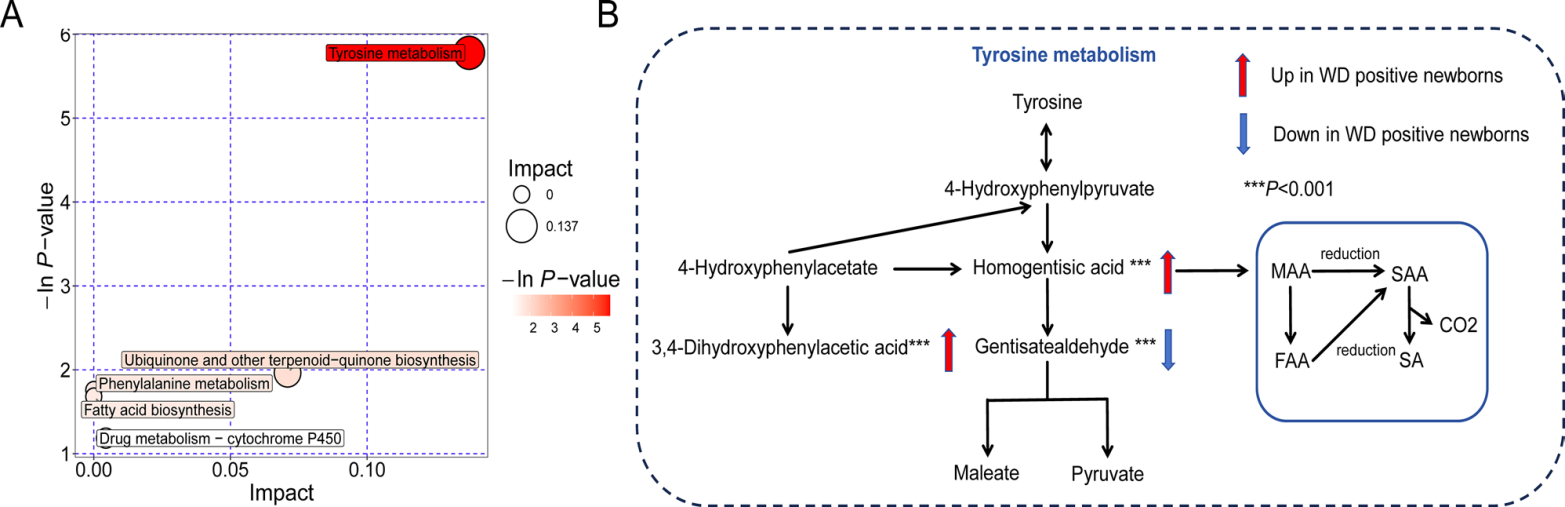
Pathway Analysis of Metabolic Alterations in WD positive newborns. (**A**) Node plot showing matched pathways according to significance (P-value) as determined by pathway enrichment analysis (y-axis), and pathways impact as determined by topology analysis (x-axis). Nodes in red indicate significance (*P* < 0.05), and the size of the nodes indicate impact. (**B**) Tyrosine metabolism pathway and main altered metabolites in this pathway. MAA, maleylacetoacetate; FAA, fumarylacetoacetate; SAA, succinylacetoacetate; SA, succinylacetone

Metabolic pathway enrichment and topology analyses were performed using KEGG and other databases. The results revealed that the tyrosine metabolism pathway was significantly enriched (*P* < 0.05) (Fig. 3A), there are a total of 76 metabolites involved in this pathway and we hit three of them— 3,4-Dihydroxyphenylacetic acid, Homogentisic acid, and Gentisaldehyde (Fig. 3B), with the first two being upregulated and the latter downregulated in the WD group.

### Predictive diagnostic model for WD

**Fig. 4:**
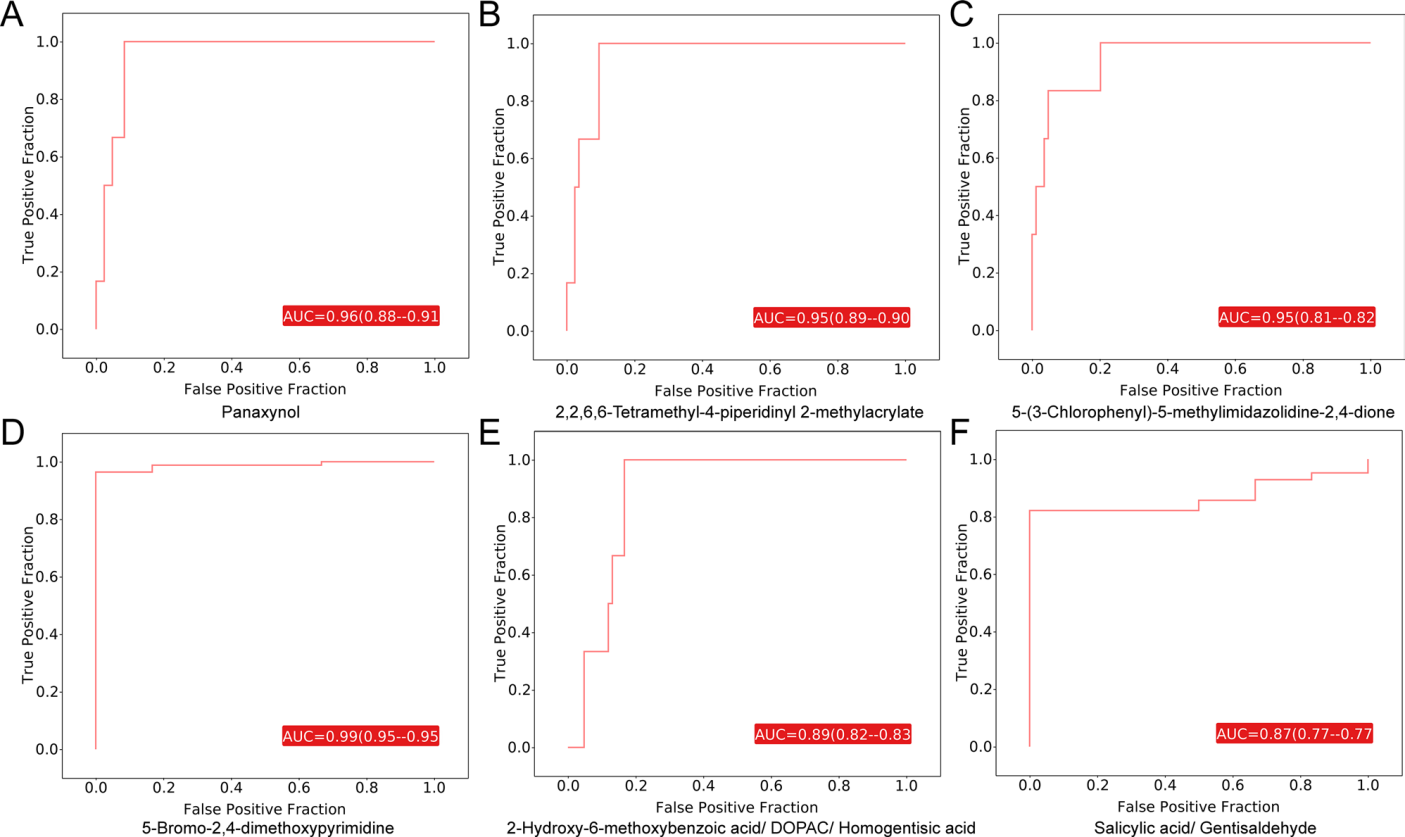
ROC curve analysis of differential metabolites. (**A**-**D**) ROC curves of differential metabolites with AUC > 0.95. (**E**) ROC curve of 2-Hydroxy-6-methoxybenzoic acid/ DOPAC/ Homogentisic acid. (**F**) ROC curve of Salicylic acid/ Gentisaldehyde

Receiver operating characteristic (ROC) curve analysis was performed for the 29 differential metabolites to evaluate their diagnostic potential for WD. All metabolites showed area under the curve (AUC) values greater than 0.70 (Table S5), indicating good discriminative ability. Among them, four metabolites exhibited excellent diagnostic performance with AUC values ≥ 0.95 (Fig. 4A-D), including three that were upregulated and one that was downregulated in the WD group. Notably, two pairs of isomeric compounds— 2-Hydroxy-6-methoxybenzoic acid, 3,4-Dihydroxyphenylacetic acid, and Homogentisic acid; Salicylic acid and Gentisaldehyde—also demonstrated strong predictive value, with AUCs of 0.89 and 0.87, respectively (Fig. 4E-F), along with high sensitivity and specificity.

## Discussion

In this study, we applied untargeted metabolomics to DBS samples from genetically confirmed WD positive and healthy newborns, identifying 29 significantly altered metabolites, including two pairs of structural isomers. Pathway enrichment analysis revealed that tyrosine metabolism was the most significantly affected pathway (*P* < 0.05). ROC analysis demonstrated that 4 metabolites had an AUC greater than 0.95, suggesting strong diagnostic potential for early detection of WD.

Metabolomics, a field that emerged in the late 20th century, investigates the changes in endogenous small-molecule metabolites to characterize physiological and pathological states [34]. In recent years, metabolomics research on WD has gained attention. Prior studies have reported altered metabolic pathways in symptomatic WD patients, including methionine metabolism, choline synthesis, the urea cycle, the TCA cycle, bile acid metabolism, lipid oxidation, and oxidative stress. Distinct metabolic biomarkers such as choline, acylcarnitines, urea, and glucose have been observed [35–38]. However, these studies were conducted in children or adults already exhibiting clinical manifestations and organ damage. Their metabolic profiles may thus reflect secondary changes due to disease progression rather than early pathophysiological events.

WD is primarily caused by copper accumulation resulting in oxidative stress, inflammation, and mitochondrial dysfunction [39]. In genetically diagnosed but asymptomatic newborns, ATP7B variants lead to impaired biosynthesis of the copper-transporting ATPase (P-ATP7B), likely inducing metabolic alterations prior to copper overload. Our study confirms that even at this presymptomatic stage, WD positive newborns exhibit significant differences in organic acids, aromatic compounds, lipids, and oxygen-containing metabolites compared with controls. These metabolic disturbances, especially in tyrosine metabolism, differ substantially from those reported in symptomatic WD patients, highlighting the potential of newborn metabolic profiling for early disease detection.

We analyzed six WD positive newborns identified through a newborn genomic screening panel. In all cases, the ATP7B variants were inherited from both parents. One newborn had decreased ceruloplasmin and abnormal liver function, indicating overt disease. The other five showed no clinical signs except for biallelic ATP7B variants; two of them had mildly reduced ceruloplasmin levels. According to the EASL Clinical Practice Guidelines [3], such individuals are classified as presymptomatic and are strongly recommended for lifelong follow-up and preventive intervention. Monitoring should include ceruloplasmin, serum copper, 24-hour urinary copper excretion, liver function tests, physical examinations focused on hepatic and neurologic signs, and slit-lamp examination for Kayser–Fleischer rings. Zinc therapy combined with a low-copper diet is the preferred treatment to prevent irreversible organ damage [40].

Interestingly, hierarchical clustering analysis (Fig. 2A) revealed that the first 15 samples in the healthy newborns group exhibited metabolic profiles resembling those of the WD-positive group. All 15 were carriers of the c.2333G > T and c.2975 C > T variants, and four of the six WD-positive cases carried these variants in trans. These findings suggest that these two variants may play a dominant role in metabolic disruption when inherited in compound heterozygosity.

Among the 29 differential metabolites, six were elevated more than 2.5-fold and five were downregulated more than 0.5-fold in the WD group. Two pairs of structural isomers were identified: (1) 3,4-Dihydroxyphenylacetic acid (DOPAC), Homogentisic acid, and 2-Hydroxy-6-methoxybenzoic acid; (2) Salicylic acid and Gentisaldehyde. Although these isomers share chemical formulas, they participate in distinct metabolic pathways. DOPAC is a downstream product of phenylalanine and tyrosine metabolism. Its upstream precursor, 3,4-Dihydroxyphenylacetaldehyde (DOPAL), is neurotoxic and promotes α-synuclein (αSyn) oligomerization, contributing to neurodegeneration [41]. The increased DOPAC in WD-positive newborns may reflect enhanced activity of aldehyde dehydrogenases (ALDH), facilitating DOPAL detoxification and offering a potential protective mechanism against early neurologic damage [42]. Notably, Cu (II) ions can exacerbate DOPAL-induced αSyn aggregation [43], which may explain the onset of irreversible neurologic symptoms once copper accumulation exceeds a critical threshold.

Homogentisic acid, when excessively elevated, deposits in connective tissues such as joints and heart valves, promoting oxidative stress and pigmentation disorders [44]. In our study, the concurrent increase in homogentisic acid and decrease in its downstream metabolite, Gentisaldehyde, may underlie the observed cardiac, renal, musculoskeletal, and dermatologic complications in WD. These disturbances further implicate tyrosine metabolism as a key pathway affected in WD newborns. Elevated homogentisic acid leads to the accumulation of maleylacetoacetate and fumarylacetoacetate, which subsequently convert to succinylacetone—metabolites known to induce hepatotoxicity and hepatocellular carcinoma in tyrosinemia type I [45]. Thus, similar mechanisms may contribute to hepatic injury in WD. Previous research has also shown that tyrosine levels are significantly lower in neurologic WD than in hepatic WD [46], potentially due to impaired dopamine biosynthesis from tyrosine, which could explain the emergence of neurologic symptoms in advanced cases [47]. Whether the newborns in this study will later develop hepatic or neurologic phenotypes associated with altered tyrosine metabolism requires long-term follow-up.

2-Hydroxy-6-methoxybenzoic acid, a methylated derivative of salicylic acid, possesses antioxidant properties [48]. In WD positive newborns, salicylic acid was decreased while 2-Hydroxy-6-methoxybenzoic acid was increased, suggesting a compensatory response to oxidative stress. Caprylic acid, a medium-chain fatty acid, has been associated with Reye-like syndrome and hepatic encephalopathy when abnormally accumulated [49]. Together, these findings suggest that dysregulated metabolites may contribute to hepatic and neurologic manifestations of WD.

Despite promising findings, this study has several limitations. First, due to the low incidence of WD, the number of positive cases was limited. Second, the inherently small volume of newborn heel-prick DBS samples precluded parallel measurement of biomarkers such as Cp and copper. Third, many metabolites remain unidentified due to incomplete database annotations or structural complexity, requiring further investigation. Additionally, the untargeted metabolomics cannot distinguish between structural isomers, potentially limiting pathway interpretation. To address these constraints, our subsequent work will focus on expanding the WD-positive cohort, implementing targeted metabolomics to validate the identified markers, and developing robust methodological frameworks for integrated analysis.

## Conclusion

Through metabolomic profiling of DBS samples from WD positive and healthy newborns, we identified a panel of significantly altered metabolites. These findings demonstrate that WD newborns exhibit distinct metabolic reprogramming prior to overt copper accumulation. Notably, we discovered potential biochemical biomarkers with promising clinical utility for early WD screening. This study not only provides a novel foundation for developing metabolite-based newborn screening strategies but also opens new avenues to improve early diagnosis and intervention for WD, ultimately mitigating disease progression.

## Supplementary Information

Below is the link to the electronic supplementary material.

Supplementary Material 1

## Data Availability

The datasets used and/or analyzed during the current study are available from the corresponding author upon reasonable request.

## Abbreviations

WD: Wilson disease
DBS: Dried blood spot
LC-MS: Liquid chromatography-mass spectrometry
ROC: Receiver operating characteristic
AUC: Area under the curve
QC: Quality control
Cp: Ceruloplasmin
K- F: Kayser-Fleischer
NGS: Next-generation sequencing
FDR: False discovery rate
OPLS-DA: Orthogonal partial least squares discriminant analysis
VIP: Variable important projection
DOPAC: 3,4-Dihydroxyphenylacetic acid
DOPAL: 3,4-Dihydroxyphenylacetaldehyde
ALDH: Aldehyde dehydrogenases
MAA: Maleylacetoacetate
FAA: Fumarylacetoacetate
SAA: Succinylacetoacetate
SA: Succinylacetone
