# Supplementary material for: Discovery of newborn Wilson disease biomarkers via integrated next-generation sequencing and untargeted metabolomics
Source: Orphanet J Rare Dis. 2025 Dec 26;21:31. doi: 10.1186/s13023-025-04173-6 (PMC12853665; doi:10.1186/s13023-025-04173-6)
Supplement: Supplementary file 1 — Supplementary Material 1 [file 13023_2025_4173_MOESM1_ESM.docx]

**Supplementary Materials**

**Supplementary tables**

Table S1. Variant sites and case numbers for the 54 carriers

| N0. | Variant Site | Case Numbers |
| --- | --- | --- |
| 1 | c.2333G>T_1 | 9 |
| 2 | c.2975C>T | 6 |
| 3 | c.1552_1553delTC | 1 |
| 4 | c.1746_1747insAA | 1 |
| 5 | c.2304_2305insC | 1 |
| 6 | c.2590_2593dupGTCA | 1 |
| 7 | c.3700delG | 1 |
| 8 | c.525_526insA | 1 |
| 9 | c.1543+1G>T | 2 |
| 10 | c.1708-5T>G | 2 |
| 11 | c.1870-2A>G | 1 |
| 12 | c.2121+2T>A | 1 |
| 13 | c.1708-1G>C, c.1168A>G | 4 |
| 14 | c.3443T>C, c.3426G>C | 1 |
| 15 | c.2975C>T, c.2333G>T | 1 |
| 16 | c.2183A>G | 3 |
| 17 | c.2605G>A | 3 |
| 18 | c.2621C>T | 3 |
| 19 | c.2755C>G | 3 |
| 20 | c.3443T>C | 3 |
| 21 | c.3532A>G | 3 |
| 22 | c.3646G>A | 3 |
|  | Total | 54 |

Table S2. Characteristics of enrolled newborns in this study

|  | WD positive newborns (n=6) | healthy newborns (n=84) | P-value |
| --- | --- | --- | --- |
| Gender(male/female) | 4/2 | 42/42 | 0.623 |
| Delivery method (Vaginal delivery/cesarean section) | 2/4 | 50/34 | 1.575 |
| Gestational Age(week) | 38.8±0.4 | 38.7±1.3 | 0.870 |
| Birthweight(g) | 3265.0±159.1 | 3336.6±420.4 | 0.681 |

Table S3. Response stability of internal standards in QC samples

|  | rt | mz | rsd |
| --- | --- | --- | --- |
| IS1 | 221.5 | 121.0444 | 0.0192 |
| IS2 | 162.5 | 133.1062 | 0.0138 |
| IS3 | 110.8 | 183.0822 | 0.0097 |
| IS4 | 30.7 | 127.0799 | 0.0292 |
| IS5 | 220.2 | 110.1078 | 0.0106 |
| IS6 | 165.2 | 127.1422 | 0.0169 |

IS, internal standard; rt, remain time; mz, Mass-to-charge ratio; rsd, Relative Standard Deviation.

Table S4. Differentially abundant metabolites in WD positive newborns compared to healthy newborns.

| **Name** | **Formula** | **Super. Class** | **MEAN healthy** | **MEAN Positive** | **VIP** | **p-value** | **FDR** | **FC** | **Log_FC** |
| --- | --- | --- | --- | --- | --- | --- | --- | --- | --- |
| Trifluoroacetic acid | C2HF3O2 | Organic acids and derivatives | 31.6801 | 6.2413 | 1.59 | <0.001 | <0.001 | 0.20 | -2.34 |
| Dimethyl ulfoxide | C2H6OS | Organosulfur compounds | 0.0849 | 0.0029 | 1.35 | <0.001 | <0.001 | 0.03 | -4.86 |
| Salicylic acid | C7H6O3 | Benzenoids | 0.0568 | 0.0286 | 1.32 | <0.001 | <0.001 | 0.50 | -0.99 |
| Gentisaldehyde | C7H6O3 | Organic oxygen compounds | 0.0568 | 0.0286 | 1.32 | <0.001 | <0.001 | 0.50 | -0.99 |
| Dehydroacetic acid | C8H8O4 | Organoheterocyclic compounds | 0.0143 | 0.0405 | 1.45 | <0.001 | <0.001 | 2.83 | 1.50 |
| 2-Hydroxy-6-methoxybenzoic acid | C8H8O4 | Benzenoids | 0.0143 | 0.0405 | 1.45 | <0.001 | <0.001 | 2.83 | 1.50 |
| 3,4-Dihydroxyphenylacetic acid | C8H8O4 | Benzenoids | 0.0143 | 0.0405 | 1.45 | <0.001 | <0.001 | 2.83 | 1.50 |
| Homogentisic acid | C8H8O4 | Benzenoids | 0.0143 | 0.0405 | 1.45 | <0.001 | <0.001 | 2.83 | 1.50 |
| Panaxynol | C17H24O | Lipids and lipid-like molecules | 0.0904 | 0.1867 | 1.49 | <0.001 | <0.001 | 2.07 | 1.05 |
| 2,2,6,6-Tetramethyl-4-piperidinyl 2-methylacrylate | C13H23NO2 | Organoheterocyclic compounds | 1.0583 | 2.1619 | 1.54 | <0.001 | <0.001 | 2.04 | 1.03 |
| 5-Bromo-2,4-dimethoxypyrimidine | C6H7BrN2O2 | Organoheterocyclic compounds | 0.0331 | 0.0223 | 1.97 | <0.001 | <0.001 | 0.67 | -0.57 |
| 2-(Dipentylamino)-2-(hydroxymethyl)-1,3-propanediol | C14H31NO3 | Organic nitrogen compounds | 0.0969 | 0.2201 | 1.32 | <0.001 | <0.001 | 2.27 | 1.18 |
| N,N-Diethyl-2-aminoethanol | C6H15NO | Organic nitrogen compounds | 0.0494 | 0.0197 | 1.10 | <0.001 | <0.001 | 0.40 | -1.33 |
| 5-(3-Chlorophenyl)-5-methylimidazolidine-2,4-dione | C10H9ClN2O2 | Organoheterocyclic compounds | 0.2793 | 0.5991 | 1.44 | <0.001 | <0.001 | 2.15 | 1.10 |
| Glabranin | C20H20O4 | Phenylpropanoids and polyketides | 0.0179 | 0.0150 | 1.70 | <0.001 | <0.001 | 0.84 | -0.25 |
| Homoarecoline | C9H15NO2 | Alkaloids and derivatives | 0.1596 | 0.3661 | 1.51 | <0.001 | <0.001 | 2.29 | 1.20 |
| Pivagabine | C9H17NO3 | Organic acids and derivatives | 0.0963 | 0.2178 | 1.46 | <0.001 | <0.001 | 2.26 | 1.18 |
| Enol-3,5,5-Trimethyl-1,2-cyclohexanedione | C9H14O2 | Organic oxygen compounds | 0.0962 | 0.1888 | 1.33 | <0.001 | <0.001 | 1.96 | 0.97 |
| N-Dodecanoyl-N-methylglycine | C15H29NO3 | Organic acids and derivatives | 0.0169 | 0.0326 | 1.49 | <0.001 | <0.001 | 1.93 | 0.95 |
| valproic acid | C8H16O2 | Lipids and lipid-like molecules | 0.1934 | 0.3373 | 1.17 | <0.001 | <0.001 | 1.74 | 0.80 |
| Caprylic acid | C8H16O2 | Lipids and lipid-like molecules | 0.1934 | 0.3373 | 1.17 | <0.001 | <0.001 | 1.74 | 0.80 |
| 5-Azauracil | C3H3N3O2 | Organoheterocyclic compounds | 0.0116 | 0.0270 | 1.39 | <0.001 | <0.001 | 2.33 | 1.22 |
| Cumyluron | C17H19ClN2O | Benzenoids | 0.0110 | 0.0279 | 1.14 | <0.001 | <0.001 | 2.53 | 1.34 |
| Phytanic acid | C20H40O2 | Lipids and lipid-like molecules | 0.0837 | 0.1146 | 1.24 | <0.001 | <0.001 | 1.37 | 0.45 |
| N2,N4-Diethyl-6-hydrazino-1,3,5-triazine-2,4-diamine | C7H15N7 | Organoheterocyclic compounds | 0.0695 | 0.1249 | 1.07 | <0.001 | <0.01 | 1.80 | 0.85 |
| Docebenone | C21H26O3 | Lipids and lipid-like molecules | 3.8631 | 6.3017 | 1.04 | <0.001 | <0.01 | 1.63 | 0.71 |
| Heptanoic_acid | C7H14O2 | Lipids and lipid-like molecules | 0.0695 | 0.0959 | 1.16 | <0.01 | <0.01 | 1.38 | 0.46 |
| Ethyl 3-(piperidin-4-yl)propanoate | C10H19NO2 | Lipids and lipid-like molecules | 0.1312 | 0.1882 | 1.09 | <0.01 | <0.01 | 1.43 | 0.52 |
| Hexadecyltrimethylammonium cation | C19H42N | Organic nitrogen compounds | 0.0833 | 0.2312 | 1.24 | <0.01 | <0.01 | 2.78 | 1.47 |
| 2-Methoxyestrone_3-glucuronide | C25H32O9 | Lipids and lipid-like molecules | 0.0102 | 0.0025 | 1.11 | <0.01 | <0.01 | 0.25 | -2.02 |
| 1,3-Dimethyl-6-(propylamino)-2,4(1H,3H)-pyrimidinedione | C9H15N3O2 | Organoheterocyclic compounds | 0.1103 | 0.2328 | 1.11 | <0.01 | <0.05 | 2.11 | 1.08 |
| SM(d18:1/18:0) | C41H83N2O6P | Lipids and lipid-like molecules | 13.2853 | 15.3070 | 1.02 | <0.05 | <0.05 | 1.15 | 0.20 |
| 2-(2,6-Difluorophenyl)-1H-benzimidazole | C13H8F2N2 | Organoheterocyclic compounds | 0.1260 | 0.3503 | 1.05 | <0.05 | <0.05 | 2.78 | 1.47 |
| 2-(4-Methoxyphenoxy)-5-nitrobenzoic acid | C14H11NO6 | Benzenoids | 0.0037 | 0.0032 | 1.10 | <0.05 | <0.05 | 0.87 | -0.21 |
| 1-(2,5-Dimethylphenoxy)-3-(4-morpholinyl)-2-propanol | C15H23NO3 | Benzenoids | 0.0584 | 0.1444 | 1.30 | <0.05 | <0.05 | 2.47 | 1.31 |
| M466T248 | C10H16N5O11PS | - | 0.0007 | 0.0005 | 1.03 | <0.05 | <0.05 | 0.70 | -0.52 |
| Glp-Trp-OEt | C18H21N3O4 | Organic acids and derivatives | 0.0205 | 0.0481 | 1.38 | <0.05 | <0.05 | 2.35 | 1.23 |
| Glaucarubin | C25H36O10 | Lipids and lipid-like molecules | 0.1012 | 0.0298 | 1.25 | <0.05 | <0.05 | 0.29 | -1.77 |
| N-Cyclohexyl-N'-[3-(trifluoromethyl) phenyl]urea | C14H17F3N2O | Benzenoids | 0.0225 | 0.0895 | 1.15 | <0.05 | <0.05 | 3.98 | 1.99 |

Only annotated metabolites are listed.Metabolites are ranked by statistical significance (smallest P-value first). VIP, Variable Importance Projection; FDR, False Discovery Rate; FC, Fold Change.

Table S5. ROC curve parameters of the 29 differential metabolites

| **No.** | **Metabolite** |  | **AUC（95%CI）**  **（%）** | **Threshold** | **Specificity（%）** | **Sensitivity（%）** |
| --- | --- | --- | --- | --- | --- | --- |
|  | **Increased** | |  |  |  |  |
| 1 | Panaxynol | | 95.6(91.3--100.0) | 0.145 | 91.7 | 100.0 |
| 2 | 2,2,6,6-Tetramethyl-4-piperidinyl 2-methylacrylate | | 95.4(90.8--100.0) | 1.647 | 90.5 | 100.0 |
| 3 | 5-(3-Chlorophenyl)-5-methylimidazolidine-2,4-dione | | 95.0(88.3--100.0) | 0.403 | 79.8 | 100.0 |
| 4 | Enol-3,5,5-Trimethyl-1,2-cyclohexanedione | | 94.2(88.8-99.7) | 0.190 | 86.9 | 100.0 |
| 5 | Homoarecoline | | 94.0(89.0--99.1) | 0.275 | 89.3 | 100.0 |
| 6 | 2-(Dipentylamino)-2-(hydroxymethyl)-1,3-propanediol | | 93.8(87.3--100.0) | 0.155 | 84.5 | 100.0 |
| 7 | Caprylic acid | | 93.3(87.8-98.7) | 0.275 | 88.1 | 100.0 |
| 8 | Hexadecyltrimethylammonium cation | | 92.5(86.7-98.2) | 0.115 | 85.7 | 100.0 |
| 9 | N2,N4-Diethyl-6-hydrazino-1,3,5-triazine-2,4-diamine | | 90.5(83.1-97.8) | 0.092 | 79.8 | 100.0 |
| 10 | 1-(2,5-Dimethylphenoxy)-3-(4-morpholinyl)-2-propanol | | 89.5(0-100.0) | 0.107 | 92.9 | 83.3 |
| 11 | Glp-Trp-OEt | | 89.5(80.8-98.2) | 0.024 | 73.8 | 100.0 |
| 12 | N-Cyclohexyl-N'-[3-(trifluoromethyl)phenyl]urea | | 89.3(0-100.0) | 0.051 | 90.5 | 83.3 |
| 13 | 2-Hydroxy-6-methoxybenzoic acid | | 88.7(81.4--96.0) | 0.034 | 83.3 | 100.0 |
| 14 | 3,4-Dihydroxyphenylacetic acid | | 88.7(81.4--96.0) | 0.034 | 83.3 | 100.0 |
| 15 | Homogentisic acid | | 88.7(81.4--96.0) | 0.034 | 83.3 | 100.0 |
| 16 | Heptanoic_acid | | 86.7(77.7-95.7) | 0.074 | 70.2 | 100.0 |
| 17 | Ethyl 3-(piperidin-4-yl)propanoate | | 86.3(74.7-97.9) | 0.163 | 79.8 | 83.3 |
| 18 | Phytanic acid | | 85.5(67.0-100.0) | 0.119 | 96.4 | 66.7 |
| 19 | 1,3-Dimethyl-6-(propylamino)-2,4(1H,3H)-pyrimidinedione | | 83.7(69.7-97.7) | 0.136 | 76.2 | 83.3 |
| 20 | 2-(2,6-Difluorophenyl)-1H-benzimidazole | | 80.8(67.6-93.9) | 0.019 | 56.0 | 100.0 |
| 21 | SM(d18:1/18:0) | | 73.2(53.4-93.0) | 0.131 | 46.4 | 100.0 |
|  | **Decreased** | | | | | |
| 22 | 5-Bromo-2,4-dimethoxypyrimidine | | 98.8(96.9--100.0) | 0.028 | 100.0 | 96.4 |
| 23 | Glabranin | | 94.8(88.1--100.0) | 0.017 | 100.0 | 82.1 |
| 24 | N,N-Diethyl-2-aminoethanol | | 89.7(80.6--98.8) | 0.030 | 100.0 | 76.2 |
| 25 | Gentisaldehyde | | 86.7(78.8--94.6) | 0.040 | 100.0 | 82.1 |
| 26 | Salicylic acid | | 86.7(78.8--94.6) | 0.040 | 100.0 | 82.1 |
| 27 | 2-Methoxyestrone_3-glucuronide | | 84.7(74.5--94.9) | 0.007 | 100.0 | 66.7 |
| 28 | Glaucarubin | | 79.6(59.7-99.6) | 0.028 | 83.3 | 79.8 |
| 29 | 2-(4-Methoxyphenoxy)-5-nitrobenzoic acid | | 71.2(43.3--99.2) | 0.003 | 50.0 | 97.6 |

ROC, Receiver Operating Characteristic curve; AUC, Area Under Curve; CI, Confidence Interval.

**Supplementary figures**


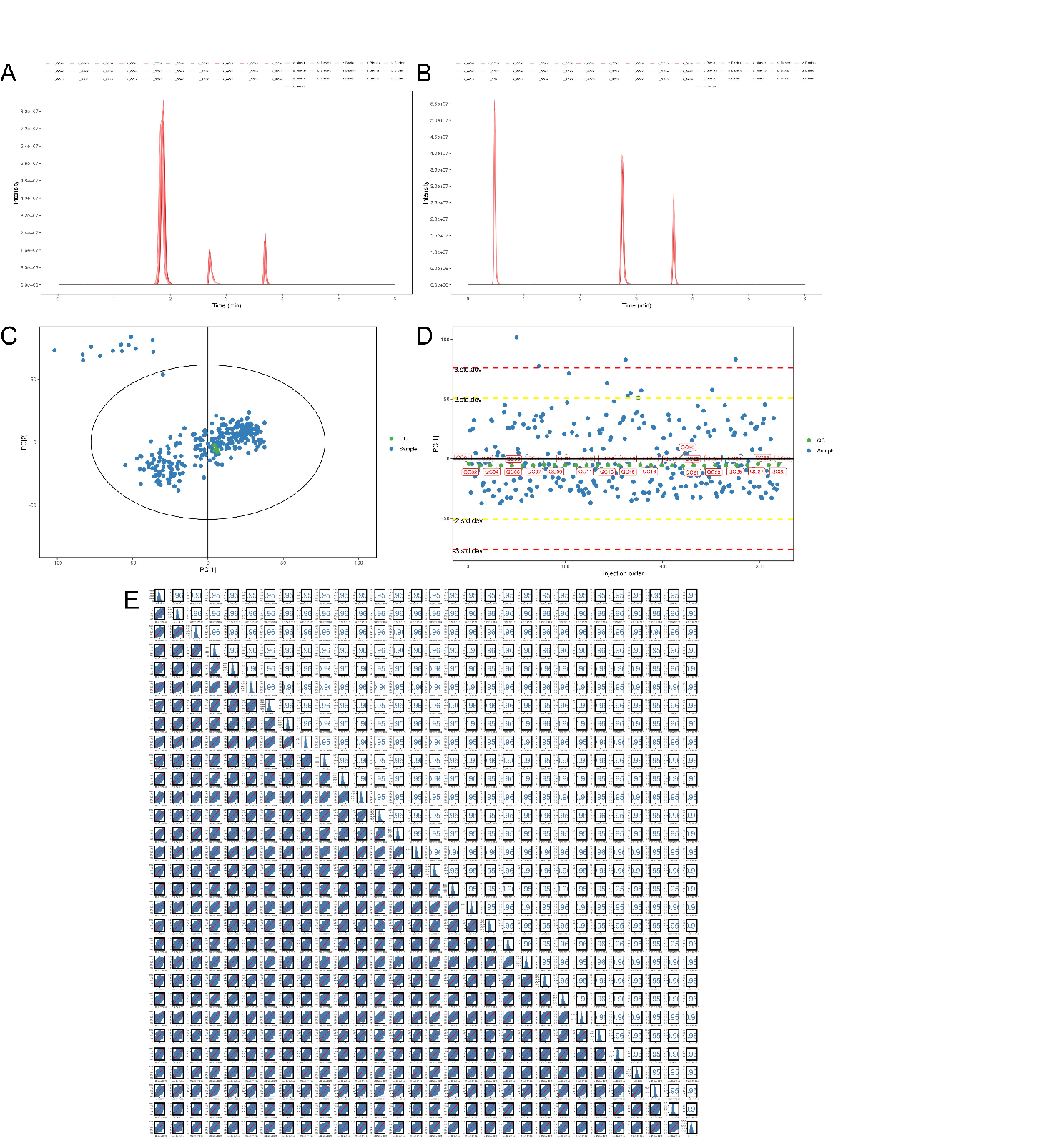


**Figure S1. Quality Control for MS/MS.** (A) Extracted ion chromatograms (EICs) of internal standards (positive mode) in QC and blank samples; (B) EICs of internal standards (negative mode) in QC and blank samples. The red line represents QC samples, and black line represent blank samples. (C) PCA score plot of the QC samples. The green dots represent the QC samples, and blue dots represent the study samples. (D) One-dimensional distribution plot of QC samples on PCA-X. (E) Correlation analysis of QC samples.

**Figure S2. Assessment of metabolite stability across storage durations.** (A) PCA model of the three negative control groups; (B) Heatmap of the top 20 differential metabolites among the groups. Neg1, negative newborns born in 2022; Neg2, negative newborns born in 2023; Neg3, negative newborns born in 2024.
